# Syneresis Behavior of Polymer Gels Aged in Different Brines from Gelants

**DOI:** 10.3390/gels8030166

**Published:** 2022-03-07

**Authors:** Hongbin Guo, Jijiang Ge, Qianhui Wu, Ziyu He, Wei Wang, Guojuan Cao

**Affiliations:** 1 School of Petroleum Engineering, China University of Petroleum (East China), Qingdao 266580, China; 15588674096@163.com (H.G.); wuqh0803@163.com (Q.W.); s18020142@s.upc.edu.cn (Z.H.); 2 Research Institute of Exploration and Development, Tarim Oilfield Company, PetroChina, Korla 841000, China; zjz19970224@163.com (W.W.); dz17667749529@126.com (G.C.)

**Keywords:** gel, HPAM, AM-AMPS, syneresis, nanoparticles

## Abstract

Gel syneresis is a common problem in gel treatment for oil recovery applications. In this study, a stable gel was prepared in a soft brine by using a water-soluble phenolic resin as a crosslinker, nanoparticles as a stabilizer, and partially hydrolyzed polyacrylamide (HPAM) or copolymers with different contents of 2-acrylamido-2-methylpropane sulfonic acid (AMPS) groups as polymers. The syneresis behavior of the gels formed in a soft brine was evaluated upon aging in hard brines. The results show that when the salinity of the hard brine is lower than 30,000 mg/L, the gel expands, and its strength decreases; when the salinity of the hard brine is higher than 50,000 mg/L, the gel exhibits syneresis, and its strength increases. The effects of various influencing factors on the gel syneresis behavior were also evaluated. It was found that optimizing the polymer structure and adding nanoparticles can effectively overcome gel syneresis and enhance gel stability. Based on the research described in this paper, some proposals for designing salt-resistant polymer gels are presented.

## 1. Introduction

Polymer gels have been used in the oil field for many years to control the flow of fluids within reservoirs [1,2]. These gels are inexpensive and easy to apply at well sites. When a gelant consisting of polymers and crosslinkers is injected into a reservoir, a viscoelastic gel will form and then create a blocked area within a period of time due to elevated reservoir temperatures. As a result, the subsequent displacement agent will bypass the blocked area, leading to a more significant sweep efficiency. Many gel systems have been suggested [2,3,4,5,6,7,8,9,10]. Polyacrylamides with various degrees of hydrolysis and molecular weights are the most common materials used to form gels by crosslinking with either metallic crosslinkers or organic crosslinkers. Nevertheless, the selected gel system should be compatible with specific conformance problems and reservoir conditions [11]. The gel must have enough stability under reservoir conditions to ensure that it can maintain good plugging performance. Otherwise, the gel may undergo processes that impair its plugging ability, such as syneresis [12,13].

Gel syneresis has previously been observed in gel-containing micromodels [14,15] and in bulk gels [16]. The main cause of syneresis is solvent expulsion from the gel, resulting in a reduction in the gel volume. Previous studies have suggested that gels exhibit syneresis after placement due to a pressure gradient. Such syneresis has been attributed to an imbalance between the forces on either side of the gel-fluid interface and related to the rigidity of the gel [17]. In fact, the gel composition and reservoir properties influence gel syneresis [18]. Crosslinking continues well past the appropriate degree once an excessive amount of crosslinker is present [19]. Depending on the composition, gels may exhibit syneresis with 5% of the initial gel volume left.

In addition, syneresis can also result from chemical modification of the polymer during aging. The acrylamide-based polymers used for water shutoff treatments are more or less prone to hydrolysis at high temperatures. The carboxylate groups produced by hydrolysis of the acrylamide groups on the polymer can further interact with divalent ions, leading to gel syneresis. This syneresis can also be considered a result of over-crosslinking between divalent ions and carboxylates [20,21]. This form of syneresis is particularly related to the use of polymers in hard brines at high temperatures.

However, there are still some cases that have been neglected. We found that many researchers developed gels using simulated brine consistent with the salinity of formation water to improve gel-reservoir compatibility. Nevertheless, gels are often not prepared with simulated brine in field applications, which would significantly increase the economic cost, but with treated oilfield effluent. Oilfield wastewater often contains water treatment agents, such as flocculants, that are difficult to clean up and may cause significant damage to the performance of the gel. Moreover, when the salinity of the water used to prepare the gel is too high, the application of many additives in the gel may be limited. For example, excessive salt can cause precipitation of some additives that enhance the gel, such as nano-silica, clay, etc. There are also some additives commonly used in gels that are difficult to dissolve in highly salinity brine. Even some oilfields do not have enough formation water or are not in a position to use formation water for gel preparation. At this point, the use of fresh water with low salinity to prepare the gel may be a better option. It is commonly believed that freshwater makes it easier to prepare gels with excellent properties, but it brings up a problem that is easily overlooked in previous studies: the difference between the salinity of the gelant and the salinity of the formation water. The polymer gels will definitely come into contact with formation water in the reservoir and the difference in the salinity between the gel and the medium surrounding the gel may also lead to syneresis of polymer gels [22,23,24]. Therefore, the effect of the difference in the salinity between the gelant and the formation water on gel dehydration deserves attention. In this study, considering the high temperature and high salt conditions, an organic crosslinker was selected to prepare gels. Commonly used organic crosslinkers include phenol, formaldehyde, hydroquinone, urotropine, and water-soluble phenolic resin, etc. [25]. Among them, water-soluble phenolic resin has low toxicity, a low price, and convenient application. Meanwhile, the gels prepared with water-soluble phenolic resin have a long gelation time, which can meet the requirements of large-dose injection. Therefore, a partially hydrolyzed polyacrylamide and two copolymers with high salinity tolerance were crosslinked with water-soluble phenolic resin to prepare the gels in a soft brine, and then their syneresis behaviors in hard brines were evaluated. The aim of this paper is to clarify the factors influencing the syneresis behavior of freshwater-prepared gels in high-salinity water and to propose methods to inhibit gel syneresis by analyzing the syneresis behavior of gels. In addition, it is hoped that the results of the study will provide effective suggestions and guidance for the design and preparation of temperature-resistant and salt-resistant gels.

## 2. Results and Discussion

### 2.1. Gelling Behavior of Different Polymers

To meet the requirement to inject a high-dose gelant, a water-soluble phenolic resin was selected as the crosslinker to prepare a gel with a long gelation time. NH_4_Cl (0.2 wt%) and SiO_2_ nanoparticles (0.2 wt%~1.0 wt%) were added to the gelant as the catalyst and stabilizer, respectively.

#### 2.1.1. Gelation Time

**Figure 1:**
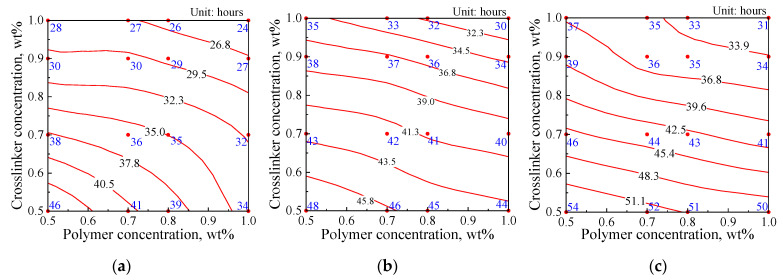
Gelation time of gels prepared with different polymers as a function of the polymer and crosslinker concentrations: (**a**) Isogram of gelation time for G3515; (**b**) Isogram of gelation time for AM-AMPS25; (**c**) Isogram of gelation time for AM-AMPS60.

**Figure 2:**
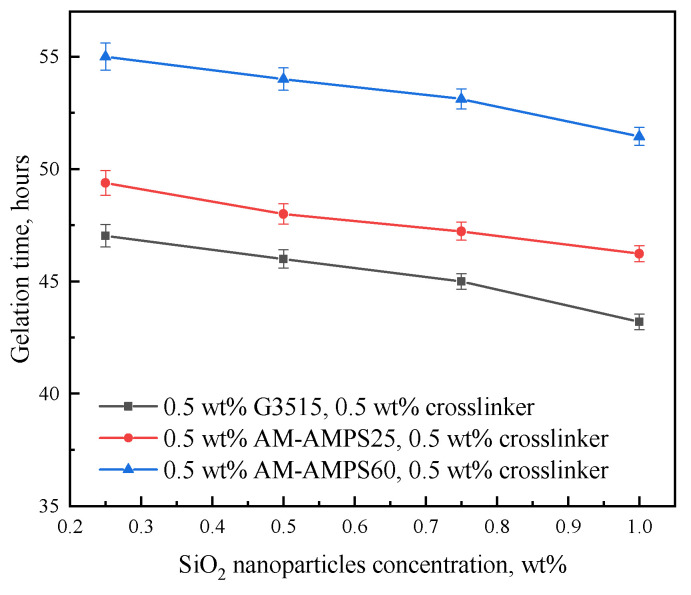
Gelation time of gels prepared with different polymers and SiO_2_ nanoparticle concentrations. (The polymer and crosslinker concentrations were fixed at 0.5 wt%).

The gelation times of gels prepared with 0.5 wt% SiO_2_ nanoparticles and different polymers are shown in Figure 1 as a function of the polymer and crosslinker concentrations. The gelation times of gels prepared with different polymers are shown in Figure 2. The polymer and crosslinker concentrations were fixed at 0.5 wt%, and the stabilizer concentration ranged from 0.25 wt% to 1 wt%.

**Figure 3:**
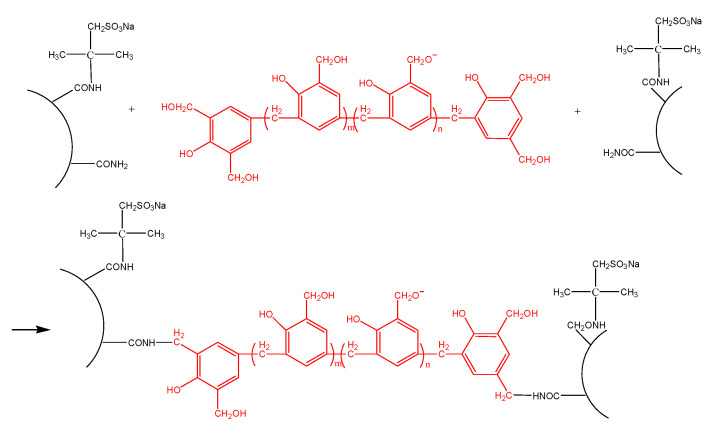
Crosslinking reaction of an AM-AMPS copolymer with water-soluble phenolic resin.

The gelation time decreased as the polymer, SiO_2_ nanoparticle, and crosslinker concentrations increased. For AM-AMPS25 and AM-AMPS60, the gelation time was longer than that of G3515 under the same polymer and crosslinker concentrations in the gelant. This result can be explained based on the condensation reaction of the amide groups on the polymer backbone with the hydroxyl methyl on the phenolic resin backbone. When the hydroxyl methyl groups are in the ortho and para positions of the phenol ring, a condensation reaction easily occurs [26]. The steric hindrances of the AMPS group on the copolymer chain and its electrostatic repulsion to the hydroxyl methyl group on the crosslinker molecule, which is electronegative, inevitably decrease the condensation reaction rate (Figure 3).

#### 2.1.2. Gel Strength

**Figure 4:**
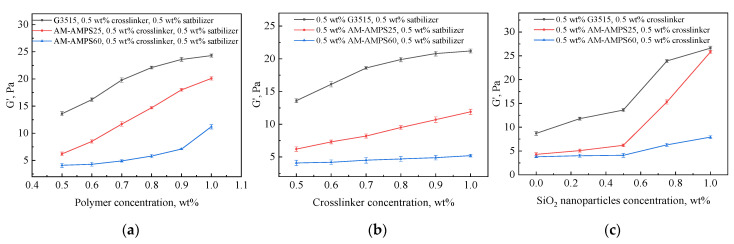
Gel storage modulus (G′) of gels prepared with different polymers. (**a**) Effect of polymer concentration on G′ of gels; (**b**) Effect of crosslinker concentration on G′ of gels; (**c**) Effect of SiO_2_ nanoparticles concentration on G′ of gels.

The storage modulus (G′) represents the temporary stored stress energy in the shear process [27], which is related to the deformation recovery capacity of the gel. In other words, the storage modulus can represent the gel strength to some extent. The effects of the polymer, crosslinker, and stabilizer on the storage modulus of gels prepared with different polymers were determined by single-factor experiments. The storage moduli of gels formulated with different components and aged at 70 °C for 7 days were determined, and the results are shown in Figure 4. Remarkably, only one of the three parameters containing polymer concentration, crosslinker concentration, and stabilizer concentration changed along the horizontal axis of the graph, while the other two concentrations remained at 0.5 wt%.

**Figure 5:**
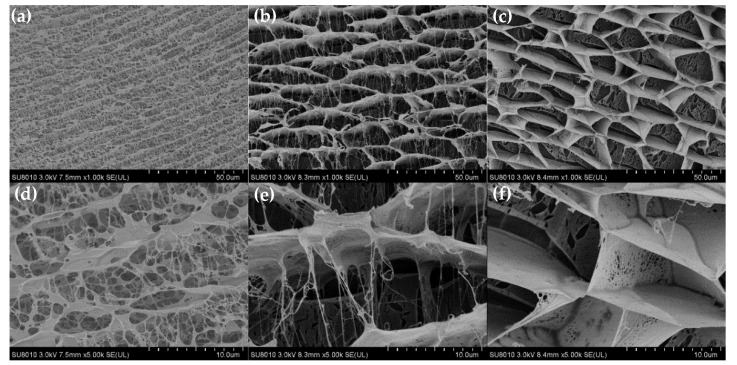
Cryo-SEM images of gels prepared with different polymers at different magnifications. (**a**) G3515 gel, ×1.00 k; (**b**) AM-AMPS25 gel, ×1.00 k; (**c**) AM-AMPS60 gel, ×1.00 k; (**d**) G3515 gel, ×5.00 k; (**e**) AM-AMPS25 gel, ×5.00 k; (**f**) AM-AMPS60 gel, ×5.00 k.

The storage modulus of the gel improved as the polymer, crosslinker or SiO_2_ nanoparticle concentrations in the gelant increased. However, for gels containing the same polymer concentration, the storage modulus of the gels prepared with AM-AMPS25 or AM-AMPS60 was always lower than that of the gels prepared with G3515. On the one hand, the relative AM content was reduced with the occurrence of AMPS groups on the copolymer chain, so the number of active points that could be crosslinked decreased, resulting in a decrease in the crosslinking density. Figure 5 shows cryo-scanning electron microscopy (cryo-SEM) images of the gels formulated with three different polymers under the same conditions. It can be clearly seen that the width of the blank area between the grids is less than 10 μm for the gels prepared by G3515, while the width of the blank area between the grids is greater than 10 μm for the gels prepared by AM-AMPS25 and AM-AMPS60. The comparison revealed that the mesh of the gel prepared by G3515 was denser than the other two polymer gels, which verified the above conjecture. On the other hand, the steric resistance effect of the AMPS group and its electrostatic repulsion to the hydroxyl methyl group on the crosslinker molecule also weakened the crosslinking reaction process.

#### 2.1.3. Thermal Stability of Gels

**Figure 6:**
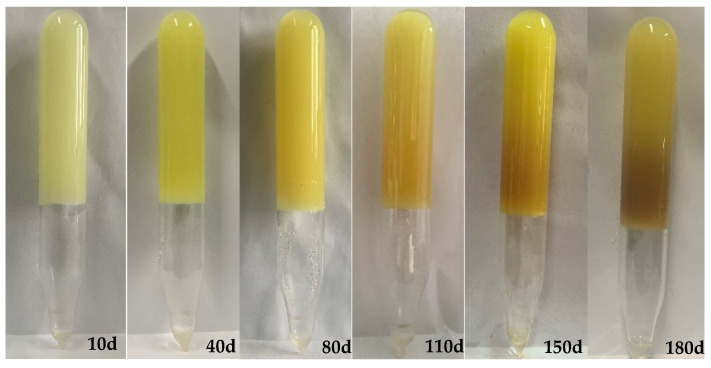
Stability of a gel strengthened by nanoparticles at 70 °C for different time.

The gel prepared with 0.5 wt% G3515, 0.5 wt% water-soluble phenolic resin, and 0.2 wt% catalyst in the soft brine underwent severe syneresis when aged at 70 °C for 3 months. To improve the stability of the gel, 0.5 wt% SiO_2_ nanoparticles were added to a gelant solution composed of 0.5 wt% G3515, 0.5 wt% water-soluble phenolic resin, and 0.2 wt% catalyst, and the obtained gel was stable at 70 °C for more than 180 days, as shown in Figure 6.

**Figure 7:**
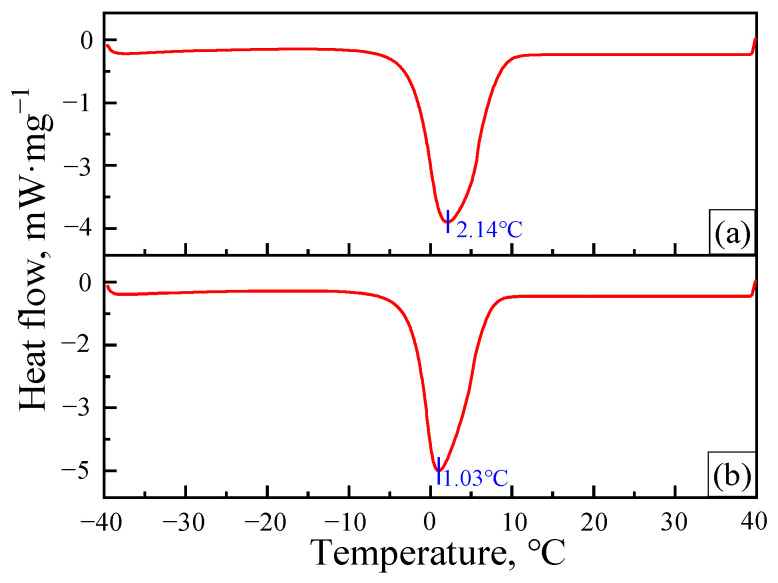
DSC curves for different gel samples: (**a**) G3515 gel without nanoparticles; (**b**) G3515 gel with 1 wt% nanoparticles.

To elucidate the mechanism by which the nanoparticles stabilized the gel, DSC measurements were used to determine the content of the different types of water in gels aged at 70 °C for 7 days in situ (Figure 7).

There are two types of water in a gel: free water and bound water [28]. Among these types of water, bonded water forms due to the strong interaction between hydrophilic groups or stabilizers and the water in the polymer gel, where its existing state in the gel is more stable [29]. A determination of the percentage of free water and bound water in a gel can represent the water holding capacity of the polymer gel and be used to characterize the stability of the gel system.

Figure 7 shows that a single endothermic peak attributed to the melting of the free water in the gel appeared at approximately −5~10 °C. By integrating the heat flow over time, the endothermic enthalpies (ΔH) for the melting of free water can be calculated during the DSC scanning process. Then, the enthalpies and percentages of free water (*w*_f_) and bound water (*w*_b_) within samples can be obtained, and the results are shown in Table 1.

**Figure 8:**
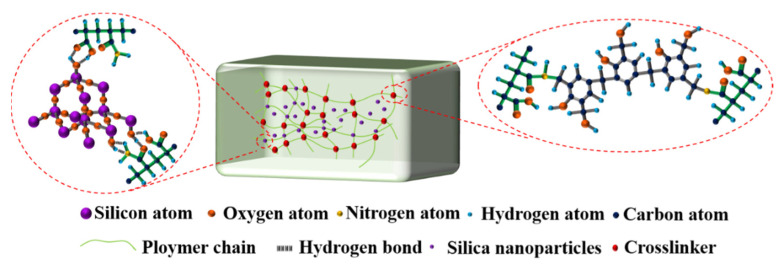
Mechanism through which nanoparticles stabilize a phenolic gel.

These results indicate that adding nanoparticles into the gelant increased the mass fraction of the bound water in the gel, which is attributed to the hydrophilicity of nanoparticles. Another reason that nanoparticles can stabilize gels may be relevant to the physical crosslinking of nanoparticles with the groups on polymer molecules. There are many hydroxyl groups on the surfaces of nanoparticles, which can form hydrogen bonds with amide groups and carboxylic acid groups on HPAM molecules, thus greatly increasing the crosslinking density of gels [30,31,32] (Figure 8).

### 2.2. Mass Changes of Gels Aged in Hard Brines

Gels are in constant contact with the formation water during gelling. Because of the salinity difference between the solvent in the gels and the formation water, gels must exhibit syneresis or swelling. Some new water channels may be formed in the blocked zone with gel syneresis, directly leading to plugging failure. Therefore, it is very important to design a stable gel under reservoir conditions.

#### 2.2.1. Effects of Crosslinker and Polymer Concentrations

**Figure 9:**
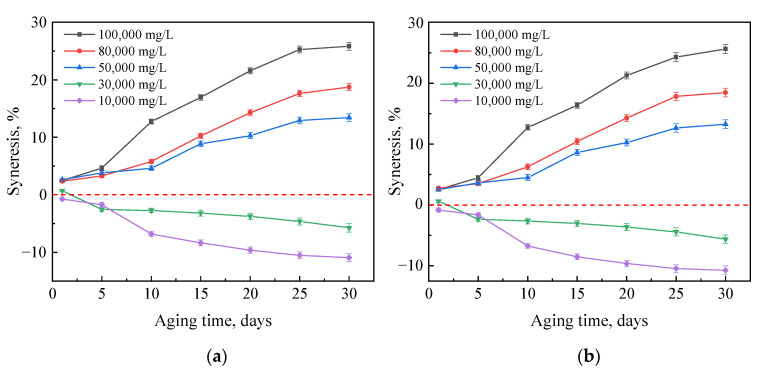

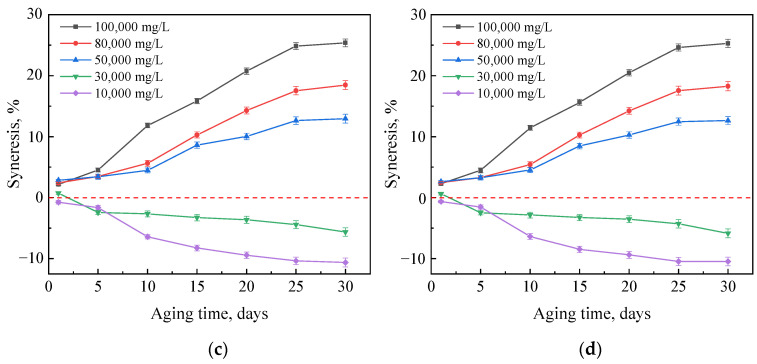
Mass changes of different gels after aging in brines with different salinities for different times: (**a**) Gels consist of 0.5 wt% G3515, 0.5 wt% crosslinker, and 0.3% stabilizer; (**b**) Gels consist of 0.5 wt% G3515, 1.0 wt% crosslinker, and 0.3% stabilizer; (**c**) Gels consist of 0.75 wt% G3515, 0.5 wt% crosslinker, and 0.3% stabilizer; (**d**) Gels consist of 0.75 wt% G3515, 1.0 wt% crosslinker, and 0.3% stabilizer.

First, a gel was prepared with 0.5 wt% G3515, 0.5 wt% water-soluble phenolic resin and 0.3 wt% nanoparticles. Then, its mass changes were investigated during gel aging in hard brines with different salinities, as shown in Figure 9a. When the salinity of the brine was less than 30,000 mg/L, the gel absorbed water and expanded. However, when the salinity of the brine was higher than 50,000 mg/L, the gel exhibited syneresis. The higher the salt content of the brine, the more serious the gel syneresis. A similar phenomenon of gel syneresis or swelling caused by salinity differences has been mentioned in previous studies [22,23].

According to polymer network theories [33,34], the degree of swelling or syneresis of a gel is governed by a balance between two potentials. One of these potentials, often referred to as the mixing potential, favors the dispersion of network chains into the solvent and, hence, causes swelling. The other potential is called the elastic potential and represents the elastic force imposed by the crosslinks that resist network chain changes from the unstrained states [35]. Because of the presence of a large number of hydrophilic groups on the polymer chain, gels tend to swell when in contact with water. As the salinity of the formation water and amide group hydrolysis increase, the carboxylic acid groups react with divalent ions such as Ca^2+^ and Mg^2+^, resulting in a decrease in the gel hydrophilicity [20]. The gel elastic potential is greater than the mixing potential, resulting in the expulsion of solvent from the gel network or syneresis. Thus, gels swell in low salinity water and exhibit syneresis in high salinity water.

When the concentration of the crosslinker and/or polymer is increased, the gel stability does not change significantly in high-salinity brines, indicating that a change in the crosslinking density does not affect the dehydration or expansion of gels, as shown in Figure 9.

#### 2.2.2. Effect of Nanoparticle Concentration

**Figure 10:**
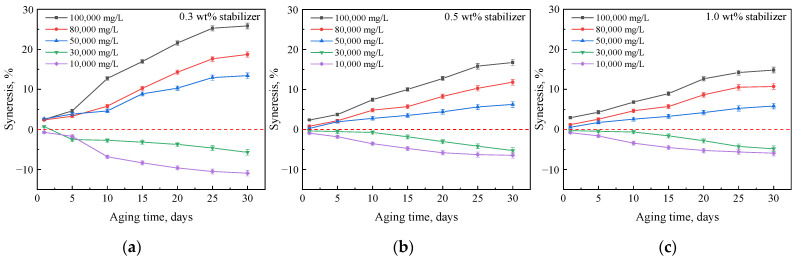
Mass changes of gels prepared with 0.5 wt% G3515, 0.5 wt% crosslinker, and different concentrations of stabilizer after aging in brines with different salinities: (**a**) 0.3 wt% stabilizer; (**b**) 0.5 wt% stabilizer; (**c**) 1.0 wt% stabilizer.

Because the salinity of the water in the reservoir is much larger than that of the gel solvent, gels may undergo syneresis when in contact with formation water. With gel shrinkage, some new water channels will appear in the blocked zone, so it is necessary to study methods to inhibit gel syneresis in brines. Gels prepared with different concentrations of silica nanoparticles were investigated during aging in hard brines, as shown in Figure 10. The results indicate that although these gels were also dehydrated in brine with salinities above 50,000 mg/L, the extent of dehydration was significantly reduced. This may be because the nanoparticles themselves were highly hydrating, and their addition increased the hydrophilicity of the gel system and improved its water-holding capacity [28,36]. In addition, the formation of hydrogen bonds between nanoparticles and amides inhibits the hydrolysis of amide groups [28,30], so the crosslinking of calcium and magnesium ions with carboxylate is also suppressed to some extent.

#### 2.2.3. Effect of Polymer Type

**Figure 11:**
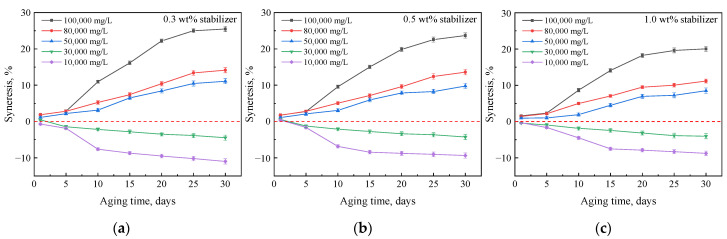
Mass changes of gels prepared with 0.5 wt% AM-AMPS25, 0.5 wt% crosslinker, and different concentrations of stabilizer after aging in brines with different salinities: (**a**) 0.3 wt% stabilizer; (**b**) 0.5 wt% stabilizer; (**c**) 1.0 wt% stabilizer.

**Figure 12:**
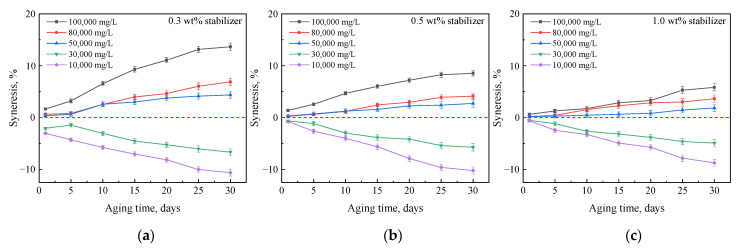
Mass changes of gels prepared with 0.5 wt% AM-AMPS60, 0.5 wt% crosslinker, and different concentrations of stabilizer after aging in brines with different salinities: (**a**) 0.3 wt% stabilizer; (**b**) 0.5 wt% stabilizer; (**c**) 1.0 wt% stabilizer.

To obtain a more stable gel, G3515 was replaced by an AM-AMPS copolymer. The results show that when the content of the AMPS group in the copolymer was low (e.g., AM-AMPS25, Figure 11), the gel stability in the brine was not much different from that of HPAM; however, the gel stability was significantly improved when the AMPS group content in the copolymer was high (e.g., AM-AMPS60, Figure 12). The degree of syneresis for gels prepared with 0.5 wt% AM-AMPS60, 0.5 wt% crosslinker, and 1.0 wt% stabilizer was approximately 5% in brine with a salinity of 100,000 mg/L and 2.5% in brine with a salinity of 80,000 mg/L.

These syneresis results may be attributable to the excellent stability of the AM-AMPS copolymer and its high salt resistance. The AMPS group has excellent thermal and salinity resistance and does not hydrolyze easily. In addition, the steric hindrance and electrostatic repulsion of the AMPS groups also inhibit the hydrolysis of the surrounding amide groups [37]. There are a large number of AMPS groups on the chain of the AM-AMPS60 polymer. Therefore, the gels prepared with AM-AMPS60 exhibited little change in hydrophilicity and were more stable in the high-salinity brines.

### 2.3. Strength Changes of Gels Aged in Hard Brines

Through the aging experiment of gel in brine, we found that the gel formed in a soft brine would absorb water and swell in low-salinity water, while it would exhibit syneresis in high-salinity brines. Although water absorption and expansion of the gel does not create new channels in the blocked formation as gel syneresis does, it does cause a decrease in the gel strength. To investigate the effect of gel expansion and syneresis on the strength of gels, the elastic moduli of gels prepared with 0.5 wt% polymer, 0.5 wt% water-soluble phenolic resin, and 0.5 wt% or 1.0 wt% nanoparticle stabilizers were tested when they were soaked in different brines for 30 days.

**Figure 13:**
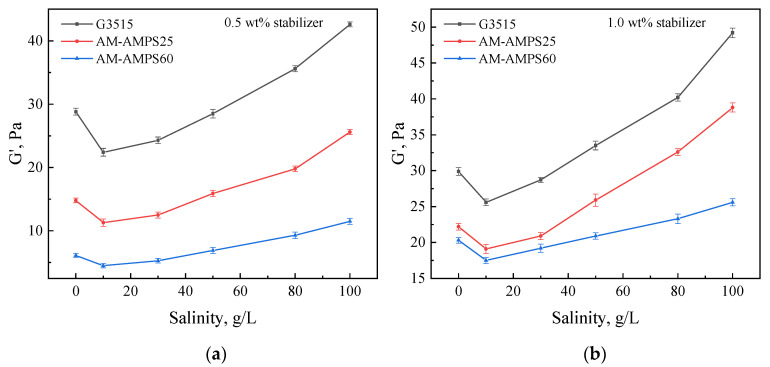
Strength changes of gels prepared with 0.5 wt% polymer and 0.5 wt% crosslinker and different concentrations of stabilizer after 30 days of aging in brines with different salinities: (**a**) 0.5 wt% stabilizer; (**b**) 1.0 wt% stabilizer.

As shown in Figure 13, the elastic modulus of the gel with 0 salt content shows that this gel only underwent aging in situ at 70 °C for 30 days. When the salinity in the water was 10,000 mg/L or 30,000 mg/L, the strength of the gel decreased because of water absorption and gel expansion. However, when the salinity was more than 30,000 mg/L, the strength of the gel increased after soaking, also corresponding to gel syneresis. In contrast, the strength variation for a gel prepared with an AM-AMPS60 polymer was relatively small in the brines.

According to the above results, we found that water absorption and expansion of the gel only slightly damaged the strength of the gels. The storage modulus of gels prepared by G3515 was still much greater than that of AM-AMPS60 gels after expansion. Therefore, the polymer should be selected flexibly, according to the salinity of the formation water in the application of gels prepared in soft brines. When the salinity of the formation water is lower than 30,000 mg/L, G3515 may be selected to prepare polymers for a higher strength. When the salinity of the formation water is higher than 50,000 mg/L, AM-AMPS60 becomes a better option for gel preparation to avoid syneresis and obtain a stable volume.

## 3. Conclusions

1. Using an AM-AMPS copolymer or HPAM as a gelatinizer, water-soluble phenolic resin as a crosslinker, and SiO_2_ nanoparticles as a stabilizer, a gel stabilized at 70 °C for more than 180 days was prepared with low salinity water.
2. When gels formed in the soft brine were aged in hard brines with salinities less than 30,000 mg/L, they absorbed water and swelled, reducing the gel strength. However, when the salinity was higher than 50,000 mg/L, the gel exhibited syneresis.
3. Optimizing the polymer structure and adding nanoparticles effectively overcame gel syneresis and enhanced the gel stability. The AM-AMPS copolymer with a high AMPS group content was more suitable for preparing salt-resistant gels.
4. The type of polymer should be flexibly selected based on the salinity of the formation water, with HPAM for salinities below 30,000 mg/L and AM-AMPS for salinities above 50,000 mg/L.

## 4. Materials and Methods

### 4.1. Materials

A water-soluble phenolic resin was obtained from Dongying Davi Technology Co., Ltd., (Dongying, China) and SiO_2_ nanoparticles with a median particle size of 7 nm were obtained from Sigma-Aldrich. Partially hydrolyzed polyacrylamide (HPAM) G3515 with a molecular weight of 14 million Daltons was obtained from Jucheng Technology Co., Ltd., Huaibei, China. Acrylamide/2-acrylamido-2-methylpropane sulfonic acid copolymers AM-AMPS25 and AM-AMPS60 were also obtained from Qucheng Technology Co., Ltd., Qingdao, China. The molecular weights of AM-AMPS25 and AM-AMPS60 were 8 million Daltons and 6 million Daltons, respectively, and their AMPS contents were 25% and 60 mol%, respectively.

In these experiments, all gelants were prepared with a soft brine, and the aging experiments were conducted in hard brines with different salinities. Table 2 and Table 3 show the compositions of the brines. The salinity of the hard brines was diluted from 100,000 mg/L to 10,000 mg/L, 30,000 mg/L, 50,000 mg/L, and 80,000 mg/L.

### 4.2. Methods

#### 4.2.1. Gel Preparation

The gelant was prepared as follows. First, 5~10 g of the water-soluble phenolic resin and 2 g of NH_4_Cl were added to a beaker with approximately 950 g of soft brine and stirred for 10 min. Next, some nanoparticles with effective concentrations ranging from 0.3% to 0.9% were separately dropped into each solution and stirred for 0.5 h. Then, 5~10 g of polymer and some soft brine water were slowly added to the above mixtures (the mass of the solution was equal to 1000 g) and stirred for 4 h to obtain the gelant. Finally, approximately 20 mL of the gelant were injected into an ampoule bottle. Each ampoule was sealed with a torch, vertically placed in a water bath, and then periodically removed to observe the gelling performance.

#### 4.2.2. Bottle Testing

The time at which a gelant starts to form a gel is important for field applications. An adequate gelation time is required for the ability to inject the gelant into the target reservoir. In this study, bottle tests were used to observe and rapidly assess the gelation time according to the Sydansk gel code [38]. The time at which the gel strength reaches grade F is called the gelation time. If the gel strength cannot reach grade F, the time required to reach the final strength of the gel is taken as the gelation time.

#### 4.2.3. Determination of Gel Strength

After the gel formed at 70 °C and aged for 7 days, the storage modulus of the gel was determined by an Anton Paar MCR92 rheometer at 25 °C. Oscillatory shear tests were conducted using a parallel plate rotor with a diameter of 25 mm and a gap of 1 mm. During the tests, a wide range of strain amplitudes was applied (0.1–1000%) at 10 rad/s.

#### 4.2.4. Gel Aged in Hard Brines

**Figure 14:**
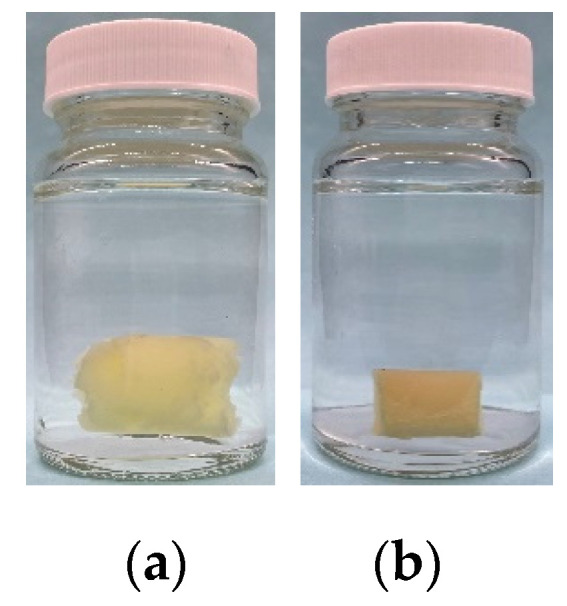
Gel aged in a hard brine with different salinities: (**a**) Brine with salinity of 10,000 mg/L; (**b**) Brine with salinity of 100,000 mg/L.

Once the gel was aged at 70 °C for 7 days in the ampoule (to distinguish it from the aging of the gel immersed in the hard brines (Figure 14), this process is called in situ aging), we broke the ampoule, carefully removed the cylindrical gel, placed it in a 100 mL screw bottle with a brine (the salt contents varied), sealed the bottle, and placed it in a water bath at 70 °C (Figure 14). Multiple samples were made for each gel, and one sample was removed at regular intervals for observation, to weigh and measure the energy storage modulus.

#### 4.2.5. Differential Scanning Calorimetry (DSC) Measurement

The adsorbed heat (Q) of freezable water was determined by a differential scanning calorimeter (TA Q200, New Castle, DE, USA). For each measurement, a nitrogen purge rate of 50 mL/min was used to control the temperature of the gel within the hermetic pan. The gel was heated from −40 °C to 40 °C, and the temperature change rates were 5 °C/min. The percentage of free water (*w*_f_) in the gel is calculated by *w*_f_ = Q/ΔH, where ΔH is the melting enthalpy of free water (333.5 J/g). Then, the bound water (*w*_b_) can be calculated by *w*_b_ = 1 − *w*_f_.

#### 4.2.6. Microstructure of Gels

The microstructure of the gel samples was observed by scanning electron microscopy (SEM) (Hitachi SU8010, Tokyo, Japan). The gel samples were lyophilized and coated with gold-palladium before being characterized by SEM. The measurements were determined at a voltage of 5 kV.

## Figures and Tables

**Table 1:** Mass fractions of bound water and free water in gel samples.

Sample	a	b
ΔH (J/g)	330.8	321.9
*w*_f_ (%)	99.19	96.52
*w*_b_ (%)	0.81	3.48

**Table 2:** Compositions of the soft brine.

Ion	Na^+^	Mg^2+^	Ca^2+^	Cl^−^	SO_4_^2−^	HCO_3_^−^	Total
Ion content (mg·L^−1^)	285	5	9	123	175	375	972

**Table 3:** Compositions of the hard brine.

Ion	Na^+^	Mg^2+^	Ca^2+^	Cl^−^	SO_4_^2−^	HCO_3_^−^	Total
Ion content (mg·L^−1^)	21,093	8141.8	8762.2	61,524	259.4	173.6	99,954

## Data Availability

The data generated or analyzed during this study are available from the corresponding author on reasonable request.
